# Combination Interventions to Prevent HCV Transmission Among People Who Inject Drugs: Modeling the Impact of Antiviral Treatment, Needle and Syringe Programs, and Opiate Substitution Therapy

**DOI:** 10.1093/cid/cit296

**Published:** 2013-08-15

**Authors:** Natasha K. Martin, Matthew Hickman, Sharon J. Hutchinson, David J. Goldberg, Peter Vickerman

**Affiliations:** 1 School of Social and Community Medicine, University of Bristol; 2 Social and Mathematical Epidemiology Group, London School of Hygiene and Tropical Medicine; 3 School of Health and Life Sciences, Glasgow Caledonian University; 4 Health Protection Scotland, Glasgow

## Abstract

***Background.*** Interventions such as opiate substitution therapy (OST) and high-coverage needle and syringe programs (HCNSP) cannot substantially reduce hepatitis C virus (HCV) prevalence among people who inject drugs (PWID). HCV antiviral treatment may prevent onward transmission. We project the impact of combining OST, HCNSP, and antiviral treatment on HCV prevalence/incidence among PWID.

***Methods.*** An HCV transmission model among PWID was used to project the combinations of OST, HCNSP, and antiviral treatment required to achieve different prevalence and incidence reductions within 10 years for 3 chronic prevalence scenarios and the impact of HCV treatment if only delivered through OST programs. Multivariate and univariate sensitivity analyses were performed.

***Results.*** Large reductions (>45%) in HCV chronic prevalence over 10 years require HCV antiviral treatment. Scaling up OST and HCNSP substantially reduces the treatment rate required to achieve specific HCV prevalence reductions. If OST and HCNSP coverage were increased to 40% each (no coverage at baseline), then annually treating 10, 23, or 42 per 1000 PWID over 10 years would halve prevalence for 20%, 40%, or 60% baseline chronic HCV prevalences, respectively. Approximately 30% fewer treatments are necessary with new direct-acting antivirals. If coverage of OST and HCNSP is 50% at baseline, similar prevalence reductions require higher treatment rates for the same OST and HCNSP coverage.

***Conclusions.*** Combining antiviral treatment with OST with HCNSP is critical for achieving substantial reductions (>50%) in HCV chronic prevalence over 10 years. Empirical studies are required on how best to scale up antiviral treatment and combine treatment with other interventions.

The global burden of liver disease caused by hepatitis C virus (HCV) is increasing faster than other causes [1, 2]. In developed countries the majority of transmissions and cases are among people who inject drugs (PWID) [1]. In the UK, PWID acquire >90% of HCV infections [3]. HCV prevalence among PWID can vary 2–3 fold within countries, with chronic prevalences of 20% in some areas up to >60% in others [4].

High-coverage needle and syringe programs (HCNSP) and opiate substitution therapy (OST) are key primary interventions, and emerging evidence suggests they can greatly reduce an individual's HCV risk [5]. Modelling has suggested that these interventions alone may not always lead to substantial reductions in HCV prevalence [6]. However, HCNSP and OST have other benefits including reducing HIV transmission [7], drug-related deaths [8], and drug-related crime [9]. In addition, there may be circumstances where their scale-up could be sufficient for achieving large reductions in HCV prevalence.

We have shown HCV treatment could have a primary role in prevention if delivered at sufficient levels to PWID [10–14] and can be more cost-effective than treating ex- or non-PWID because of prevented secondary infections [15]. We consider the impact of combining OST, HCNSP, and HCV treatment on HCV prevalence and incidence among PWID.

## METHODS

### Model Description

We extended our dynamic, deterministic model of HCV transmission and treatment among PWID [10] to include movement of PWID through various intervention states (OST and HCNSP, defined as obtaining 1 or more sterile syringes from an NSP for each injection). The model schematic and equations can be found in the Supplementary material.

Briefly, all PWID are initially susceptible (*X_j,k_*, where subscripts represent intervention coverage such that off/on HCNSP [*j* = 0 or 1, respectively] and off/on OST [*k* = 0 or 1, respectively]) and become HCV infected at a per-capita rate, λ_j,k_, specific to that intervention state. A proportion (δ) of those acutely infected will spontaneously clear infection and be at risk of reinfection (*E_j,k_*), while the remainder (1 − δ) proceed to chronic infection, *C_j,k_*. Chronically infected PWID can be put on antiviral treatment (T_*j,k*_) at a rate of Φ per 1000 PWID annually for a duration 1/ω, whereupon a proportion (α) attain sustained viral response (SVR) and move to the previously infected compartment, *E_j,k_*, where they are at risk of reinfection. Those who do not attain SVR (1 − α) move to the treatment failure compartment, *F_j,k_*, where we assume they cannot be retreated. PWID leave all stages through permanent cessation of drug use (μ_1_) or death due to drug- or nondrug-related causes (μ_2_). The model does not include an acute infection category due to their small probable contribution (<2%) to HCV transmission even if they have heightened viremia [14] and also ignores any immunity following treatment or spontaneous clearance. Immunity is neglected in part because the evidence is uncertain [16] but also because previous analyses suggested that incorporation of partial immunity has negligible impact [6, 10].

PWID are tracked through 4 intervention states: no intervention, OST only, HCNSP only, and OST with HCNSP. All PWID initially enter the no-intervention state. We assume the per-capita recruitment rates to OST (β) and HCNSP (η) are independent of the current intervention state [6]. The rates of leaving OST and HCNSP are γ and κ, respectively.

The forces of infection for each susceptible state were defined by the relative risk in that state, such that infectivity and susceptibility were altered by a factor Γ, Π, or Β if the PWID was on OST, HCNSP, or both, respectively. The chance of a PWID having a transmission event with any PWID from another risk state and infectious status was assumed to be proportional to the relative frequency of transmission events for PWID in that state. Due to rapid reductions in viral load while on antiviral treatment, we assume the transmission potential of those on treatment is scaled down by a factor depending on the SVR rate.

### Intervention Parameters

Model parameters can be found in Table 1. Effect estimates for PWID on OST or HCNSP were taken from a pooled analysis of UK data [5, 6]. We use a pooled SVR rate for pegylated interferon (pegIFN) and ribavirin (RBV) from a meta-analysis of individuals who report actively injecting (median 61.4%, range 51.2%–71.6%) [17] and explore the impact of new IFN-free direct-acting antiviral (DAA) treatments, using SVR rates from phase II studies (median 90%, range 80%–100%) [18–20].

**Table 1.** Model Parameters and Sources

Parameter	Symbol	Value(s) or Range	Units	References
HCV chronic prevalence^a^	Vary π to fit	20%, 40%, or 60%	…	…
PWID population size	Vary θ to fit	1000	…	…
Exit rate (cessation + death)	μ_1__+_ μ_2_	8.5%	per year	As in [6, 10], sensitivity analysis varied 5%–20% per year [21, 26–28]
Recruitment rate on OST	β	(0%–55%)	per month	Varied to achieve a range of intervention coverages
Recruitment rate on HCNSP	η	Set equal to recruitment rate on OST	per month	Varied to achieve a range of intervention coverages
Duration on OST	12/γ	8	months	[6, 8]
Duration on HCNSP	12/κ	8	months	[6] Few data, assumed the same as OST
Proportion spontaneously clear	δ	25%	…	[29]
Annual PWID treatment rate	Φ	0–100	per 1000 PWID	Varied to achieve a range of intervention coverages
PEG-IFN + RBV SVR	α	61.4% (51.2%–71.6%)	…	[17]Sampled from a uniform distribution
IFN-free DAA SVR	α	90% (80%–100%)	…	[18–20]Sampled from a uniform distribution
PEG-IFN + RBV duration	52/ω	24	weeks	[30]
IFN-free DAA duration	52/ω	12 (8–16)	weeks	[18–20] Sampled from a uniform distribution
Relative risk for acquiring HCV on OST	Γ	0.48 (0.17–1.33)	…	[5, 6] Sampled from a lognormal distribution
Relative risk for acquiring HCV on HCNSP	Π	0.50 (0.22–1.12)	…	[5, 6] Sampled from a lognormal distribution
Relative risk for acquiring HCV on OST and HCNSP	Γ	0.21 (0.08–0.52)	…	[5, 6] Sampled from a lognormal distribution.

Abbreviations: DAAs, direct-acting antivirals; HCNSP, high-coverage needle and syringe programs, defined as receiving 1 or more sterile syringes from an NSP per injection per month; OST, opiate substation therapy; peg-IFN, pegylated interferon; PWID, people who inject drugs; RBV, ribavirin; SVR, sustained viral response.

^a^ Used to estimate the infection rate, π (vary π and fit to the hepatitis C virus chronic prevalence).

### Modeled Scenario Analysis

We project the 10-year impact on HCV prevalence and incidence for various combinations of scale-up of antiviral treatment (from none at baseline), OST and HCNSP (from 0%, 20%, or 50% coverage of each at baseline) for 3 baseline HCV chronic prevalence settings (20%, 40%, and 60%). We explore the impact of treatment using peg-IFN + RBV or new IFN-free DAAs in combination with scale-up of OST and HCNSP to a maximum of 80% for each. To do this, we performed a multivariate uncertainty analysis by running the model with 1000 randomly sampled parameter values from the uncertainty distributions for the antiviral treatment SVR and the efficacy of OST and HCNSP on reducing HCV transmission risk (Table 1). We utilize the projections to determine combinations of antiviral treatment, OST and HCNSP scale-up that halve baseline chronic prevalence within 10 years. Contour maps show what prevalence and incidence reductions are achievable with various levels of scale-up of antiviral treatment, OST and HCNSP using median estimates for the efficacy of OST, HCNSP, and SVR.

#### HCV Treatment Delivered Within OST Programs

Since HCV antiviral treatment may best be delivered to PWID alongside OST, we explore the impact of restricting treatment to only those on OST. We calculate the minimum coverage of OST and HCNSP required to achieve different relative prevalence reductions for 20%, 40%, and 60% HCV chronic prevalence scenarios with no interventions at baseline. Model projections assume median estimates for efficacy of OST, HCNSP, and SVR for peg-IFN + RBV and assume either all chronically infected PWID on OST are treated annually (limited by the HCV prevalence) or 5% of PWID on OST are treated annually.

### Sensitivity Analysis

We perform a 1-way sensitivity analysis on the intervention combinations required to halve baseline chronic prevalence over 10 years for the 40% baseline prevalence scenario with no baseline OST or HCNSP. Model projections assume median estimates for efficacy of OST, HCNSP, and SVR for peg-IFN + RBV (Table 1) and explore the impact of including PWID risk heterogeneity and varying the exit rate (due to injecting cessation or death). For the risk heterogeneity sensitivity analysis, we simulate a high-risk population comprised of 50% of PWID (the remainder low risk), no turnover between high- and low-risk states, and increased HCV risk among the high-risk group of 2- or 6-fold that of the low-risk PWID. We also explore scenarios where the high- and low-risk groups mix proportionally or partially (50%) assortatively and the effect of assuming that a proportion (20%) of PWID never go on OST.

## RESULTS

### Scaling Up From Baseline With peg-IFN + RBV

**Figure 1.**
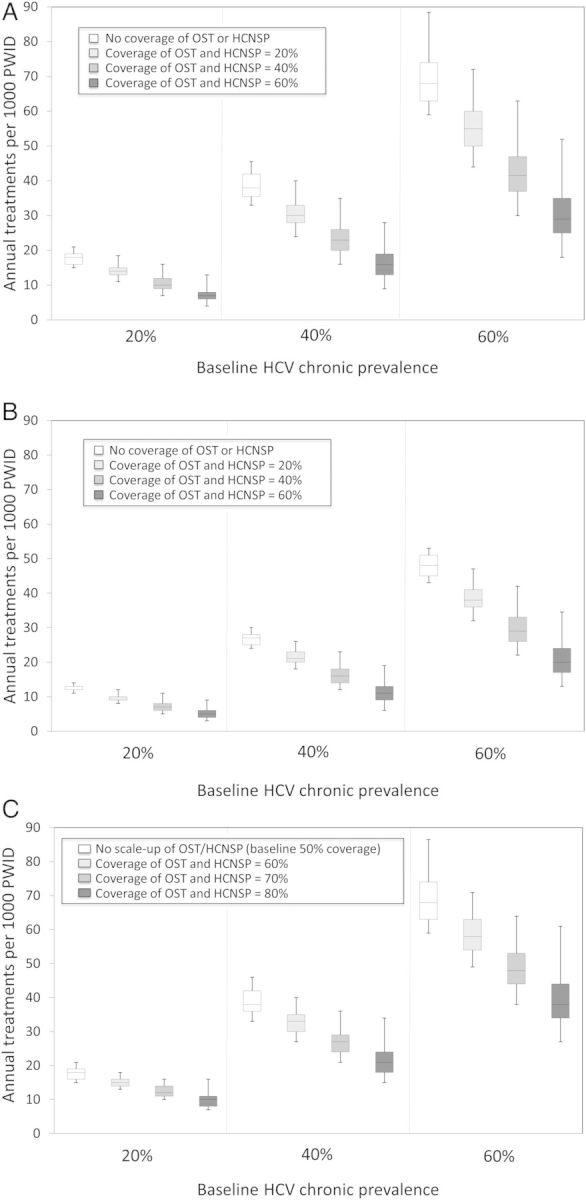
Combinations of annual treatment rates per 1000 injectors and coverage of opiate substitution therapy (OST) and high-coverage needle and syringe programs (HCNSP) required to reduce prevalence by 50% within 10 years. Results shown for 3 baseline chronic prevalence settings (20%, 40%, and 60%). *A* and *B*, Assumes no intervention coverage at baseline with OST and HCNSP scale-up to 0%, 20%, 40%, or 60% of each and using pegylated interferon and ribavirin (peg-IFN + RBV) (*A*) and interferon (IFN)-free direct-acting antivirals (DAAs) (*B*). *C*, Assumes 50% coverage of OST and HCNSP at baseline with OST and HCNSP scale-up to 50%, 60%, 70%, or 80% of each using peg-IFN + RBV. The box-plots signify the uncertainty (middle line is the median, limits of the boxes are 25% and 75% percentiles and whiskers are 2.5% and 97.5% percentiles) in the impact projections due to uncertainty in the intervention effect estimates.

For a baseline chronic HCV prevalence of 20%, 40%, or 60%, Figure 1*A* shows that by combining interventions (involving peg-IFN + RBV), chronic prevalence can halve within 10 years. The model projections vary considerably (95% credible interval [CrI] deviates 6%–21% from median projections for treatment only, with increasing uncertainty including OST and HCNSP scale-up [24%–71% deviation with 60% scale-up of OST and HCNSP]). Scaling up OST and HCNSP will substantially decrease the treatment rate required to halve prevalence within 10 years (by 19%–27% or 39%–44%, respectively). Hence, if coverage of OST and HCNSP were both increased to 40%, then annually treating 10 (95% CrI, 8–14), 23 (95% CrI, 19–32), and 42 (95% CrI, 35–58) per 1000 PWID would halve prevalence over 10 years in the 20%, 40%, or 60% chronic HCV prevalence scenarios, respectively, as compared to treating 18 (95% CrI, 17–20), 38 (95% CrI, 36–42), and 68 (95% CrI, 64–83) per 1000 PWID annually with no OST or HCNSP coverage.

**Figure 2.**
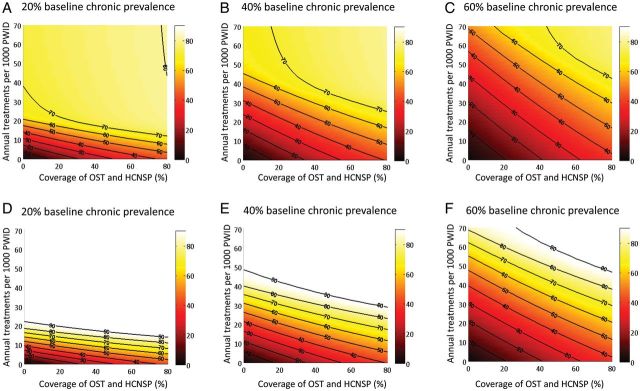
Contour maps of the relative reductions in prevalence (%) at 10 years with combinations of antiviral treatment (*y-*axis) and opiate substitution therapy/high-coverage needle and syringe program (OST and HCNSP) (*x*-axis) scale-up with no baseline coverage of OST, HCNSP, or treatment. Results shown for 3 baseline hepatitis C virus chronic prevalence settings (20%, 40%, and 60%) with pegylated interferon and ribavirin (pegIFN + RBV) (*A*–*C*) and IFN-free direct-acting antivirals (*D*–*F).* Projections used the median estimates for efficacy of OST, HCNSP, and peg-IFN + RBV from Table 1.

Contour maps of the relative prevalence reductions for various combinations of intervention scale-up over 10 years show that scale-up of OST and HCNSP reduces the required treatment rate necessary to achieve a given impact (Figure 2) and that HCV treatment is required to achieve >45% reduction in prevalence within 10 years.

For a given coverage of OST and HCNSP, greater relative reductions in incidence are achieved at 10 years than for prevalence, with the relative impact on incidence being 101%–130% greater than the impact on prevalence for OST and HCNSP scale-up to 60% coverage (Supplementary Figure 2). In contrast, treatment alone has equal impact on incidence as prevalence.

### Scaling Up From Baseline With IFN-free DAAs

Results simulating the likely impact of new IFN-free DAAs are shown in Figures 1*B* and 2*D*–*F* and suggest that approximately 30% fewer treatments are necessary than with peg-IFN + RBV to halve prevalence within 10 years. Increasing OST and HCNSP coverage to 40% may only require annual treatment rates of 7 (95% CrI, 6–10), 16 (95% CrI, 14–21), and 29 (95% CrI, 25–39) per 1000 PWID for 20%, 40%, or 60% baseline chronic prevalences, respectively, to achieve a halving of prevalence in 10 years.

### Scaling Up From 20% or 50% Baseline Coverage of OST and HCNSP With peg-IFN + RBV

Figure 1*C* shows the required levels of intervention scale-up necessary for halving chronic prevalence within 10 years with 50% OST and HCNSP at baseline. For example, prevalence can be halved within 10 years by increasing OST and HCNSP coverage from 50% to 70% and annually treating 12 (95% CrI, 11–14), 27 (95% CrI, 24–34), and 48 (95% CrI, 42–59) per 1000 PWID for the 20%, 40%, or 60% chronic prevalence scenarios, respectively. Scaling up OST and HCNSP from already moderate or high coverage levels also leads to greater reductions in the number of antiviral HCV treatments required to achieve chronic HCV prevalence reductions as compared to scale-up from no OST or HCNSP at baseline. At 20% coverage of OST and HCNSP at baseline, achieving >40% prevalence reduction within 10 years always requires scale-up of antiviral treatment, whereas at 50% baseline coverage, treatment is always required to achieve >30% prevalence reductions at 10 years (Supplementary Figure 3).

### Scaling Up HCV Treatment Through OST Programs

**Figure 3.**
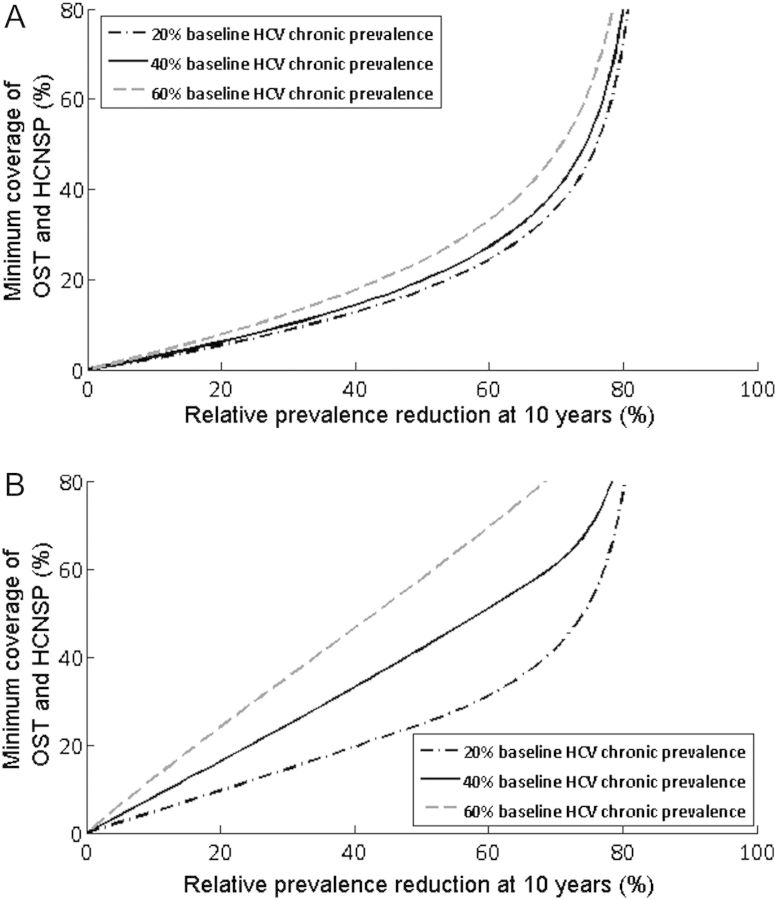
Minimum coverage of opiate substitution therapy/high-coverage needle and syringe programs (OST and HCNSP) required if antiviral treatment (pegylated interferon and ribavirin [peg-IFN + RBV]) is delivered alongside OST. Figures show the minimum coverage of OST and HCNSP required (*y*-axis) for various desired relative prevalence reductions at 10 years (*x*-axis) with the 20%, 40%, and 60% baseline hepatitis C virus (HCV) chronic prevalence settings if all infected people who inject drugs (PWID) on OST are treated annually, limited by HCV prevalence (*A*) or 5% of PWID on OST are treated annually (*B*). Projections used the median estimates for efficacy of OST, HCNSP, and peg-IFN + RBV from Table 1.

Figure 3 shows the minimum coverage of OST and HCNSP required (no coverage at baseline) to achieve different relative reductions in prevalence at 10 years, while assuming either there are no limits to treatment capacity/uptake within OST (all infected PWID on OST are treated each year) or that 5% of PWID on OST are treated annually. If all infected PWID on OST can be treated annually, halving prevalence in 10 years requires 18%–24% coverage of OST and HCNSP, whereas higher coverage levels are required if only 5% of PWID on OST are annually treated (25%–58% OST and HCNSP).

### Sensitivity Analysis

At higher exit rates (ie, in populations with shorter durations of injecting) scaling up OST and HCNSP achieves more impact than at lower exit rates, whereas the opposite occurs for scaling up antiviral treatment, but less so (Supplementary Figure 4*A* and *B*). Therefore, to halve prevalence within 10 years, a strategy using treatment alone would require more treatments at a high exit rate (shorter injecting duration) than at a low exit rate (longer duration), but a strategy using just OST and HCNSP would require the opposite (Supplementary Figure 4*A*). To minimize the uncertainty around injecting duration, it is possible to choose an intervention combination that achieves the same impact regardless of exit rate (Supplementary Figure 4*C*).

The model projections are insensitive to the inclusion of a high-risk group, even if it comprised 50% of the population with no turnover between high and low risk (Supplementary Figure 4*D*). Only if the high-risk group has a 6-fold relative risk and mixes partially assortatively with no turnover does the required number of treatments increase by a noticeable degree (20%–35% for a given OST and HCNSP coverage). In contrast, if there is turnover between risk groups, then heterogeneity has little effect. Additionally, there is no difference in the required treatment rates if 20% of the population never go on OST.

## DISCUSSION

We projected the impact of combining OST, HCNSP, and HCV antiviral treatment on HCV prevalence and incidence among PWID. Halving chronic HCV prevalence within 10 years is not possible using OST and HCNSP alone but is achievable in all prevalence settings when combined with current treatments (peg-IFN + RBV) and will be more achievable with new IFN-free DAAs. For a given coverage of OST and HCNSP, greater reductions in incidence are achieved at 10 years than for prevalence, whereas no difference is found with antiviral treatment. This is because OST and NSP directly reduces incidence, while treatment directly reduces prevalence by curing infections. In general, increasing coverage of OST and HCNSP by 20% from any level reduces the required number of treatments by about 30%. In settings with shorter average injecting durations (such as South East Asia), scale-up of OST and HCNSP may be preferable as there is little time for the benefits of antiviral treatment to accrue. Conversely, in areas with long injecting durations (such as Zurich [21]), OST and HCNSP impact will be much reduced, so treatment is critical for achieving substantial HCV reductions. Finally, heterogeneity in injecting risk has marginal impact in most realistic scenarios.

### Limitations

These projections are based on a theoretical model with several limitations. First, there is uncertainty in the model parameters including efficacy estimats for OST and HCV, and HCV antiviral treatment SVR rates among PWID. Second, it remains to be demonstrated that the higher treatment rates projected in some of the scenarios can be achieved, although the new IFN-free DAA treatment should make scale-up easier to implement, if earlier trials suggesting shorter treatment duration, higher SVR and lower toxicity than current treatment regimes [20] prove to be true. Multivariate sensitivity analyses were used to explore the implications of parameter uncertainty, including wide sampling ranges around the SVR estimates to account for settings with different SVR rates or genotype distributions.

In addition, complexities involved in scaling up each intervention were not considered here. Previous modeling analyses considered these issues for OST and HCNSP [6], but additional case-finding interventions may be required.

Third, we neglect the other benefits of OST and HCNSP, such as the impact on reducing HIV transmission [7], drug-related deaths [8], and drug-related crime [9]. These benefits would accrue in addition to any HCV benefits and so give added impetus to scaling up HCNSP and especially OST.

### Implications

Overall, our work supports current recommendations on HCV prevention issued by the European Centre for Disease Prevention and Control that interventions be combined to achieve maximum impact [22], as well as previous modeling studies that have shown that scale-up of antiviral treatment [10–14, 23], OST and HCNSP [6] among PWID can reduce prevalence in a variety of chronic prevalence settings. However, the relative affordability of each strategy is a key question, particularly with the new DAAs. Current treatment with peg-IFN + RBV costs between $16 000 and $33 000 per full treatment course, whereas triple therapy with boceprevir and telaprevir costs approximately $30 000–$80 000 [24]. By contrast, annual costs of delivering OST have been estimated at $10–$15 per day ($3650–$5475 per year) and high-coverage NSP may cost ∼$500 per year [25]. Therefore, strategies that increase OST and HCNSP in order to minimize the number of antiviral HCV treatments required to prevent and reduce chronic HCV are likely to be an efficient use of resources. Future work should address the optimal combination prevention intervention strategy that addresses both cost effectiveness and affordability.

## Supplementary Data

Supplementary materials are available at *Clinical Infectious Diseases* online (http://cid.oxfordjournals.org/). Supplementary materials consist of data provided by the author that are published to benefit the reader. The posted materials are not copyedited. The contents of all supplementary data are the sole responsibility of the authors. Questions or messages regarding errors should be addressed to the author.
